# Thyroid auto immune antibodies in children with Type-I Diabetes mellitus in relation to diabetes control

**DOI:** 10.12669/pjms.35.4.192

**Published:** 2019

**Authors:** Muneera Fadhil Ridha, Munib Ahmed Al Zubaidi

**Affiliations:** 1 Muneera Fadhil Ridha, Department of Pediatrics, University of Baghdad, College of Medicine, Baghdad, Iraq; 2 Munib Ahmed Al Zubaidi, Department of Pediatrics, University of Baghdad, College of Medicine, Baghdad, Iraq

**Keywords:** Autoimmune thyroid disease, Diabetic control, Type 1 diabetes mellitus

## Abstract

**Background & Objective:**

As an autoimmune disease, Type-1 diabetes mellitus (DM) may be associated with other autoimmune disorders, the presence of thyroid antibodies could be negatively impact the diabetic control. Our objective was to investigate thyroid autoimmunity in a cohort of children and adolescents with Type-1 diabetes and the Influence of the presence of thyroid autoimmune abnormalities on the control of diabetes in group of Iraqi pediatric patients with Type-I D.M.

**Methods:**

This study was conducted at the Medical City Complex, Children Welfare Hospital, Baghdad, Iraq. This study was carried out from the first of January 2016 till the end of September 2017. Data were analyzed from 150 patients with Type-1 diabetes, aged 1–18 years who were treated and are coming for regular follow up in the diabetic clinic. Thyroid functions tests, Antibodies to thyroglobulin (anti-TG) and thyroperoxidase (anti-TPO) were measured, documented and correlated with diabetic control according to glycated haemoglobin (HbA1c) level.

**Results:**

In the total of 150 patients, positive Antibodies to thyroglobulin (anti TG) were more in ≤3 years duration group of Diabetes mellitus(DM) and negative anti TG was less in the >3 years duration of DM group with statistically significant results (p=0.043), Regarding the distribution of thyroid antibodies (AB) according to HbA1c group, there was progressive positive anti thyroperoxidase (anti TPO) titer with glycemic status, good glycemic control had the lowest positive anti TPO titer and poor glycemic control group had the highest positive anti TPO titer and the result was statistically significant (p=0.048).

**Conclusions:**

Thyroid autoimmunity may be associated with poor diabetic control and elevated TSH levels, indicating subclinical hypothyroidism that my affect the diabetic control.

## INTRODUCTION

Type-I diabetes mellitus is an auto immune disease.^1^ It can be associated with other auto immune disorders that may influence the control of diabetes by disruption the function of respective organs.^1^

The prevalence of positive thyroid antibodies in children with Type-1 diabetes varies considerably between 3 and 50% in different countries^2^, and the clinical significance of these antibodies remains controversial. Moreover, there has been no consensus on screening for autoimmune thyroiditis in patients with Type-1 diabetes.^3^

Auto immune thyroid disease (AITD), is the most common auto immune disease associated with type I diabetes mellitus, The incidence is expected to be 2–4-folds higher than in general population^4^, as Hashimoto’s thyroiditis is the most common clinical form (14–28%), the Graves’ disease my noticed less frequently (0.5–7%).^5^

The screening and diagnosis of AITD are based on the assessment of autoantibodies to thyroid peroxidase (anti-TPO) and thyroglobulin (anti-TG). The prevalence of these autoantibodies is dependent on gender, age of patient, and age at the onset of diabetes. It also varies in different geographic regions and is known to be higher in regions with higher iodine intake.^6^,^7^

The aim of this study was to evaluate the thyroid autoantibody profile in Type-1 diabetic patients and the Influence of the presence of thyroid autoimmune abnormalities on the control of diabetes in group of Iraqi pediatric patients with Type-I D.M.

## METHODS

This study was conducted at the Medical City Complex, Children Welfare Hospital, Baghdad, Iraq. This study was carried out from the first of January 2016 till the end of September 2017. One hundred fifty children and adolescents aged during (1 year – 18 years) with Type-1 diabetic patients coming for regular follow up in the diabetic clinic were randomly selected after taking an informed consent were included in this study. Detailed history was taken from the patients and their parents and the following data were collected: Name of the patient, contact telephone number, age (date of birth), sex, date of diagnosis of diabetes and age at diagnosis and duration of diabetes were calculated and family history of any other auto immune diseases. Glycated hemoglobin A. was estimated by using fast ion-exchange Resin Separation Method. The glycated haemoglobin (HbA1c) results for the last one year and the mean for which was calculated and considered as an indicator for glycemic control as the following: (HbA1c =6-7.9) Good control, (HbA1c=8-9.9) Fair control and (HbA1c>10) poor control.^8^ Thyroid function test was measured as following: Two ml were taken and sent for estimation of (T3,T4 and TSH). Normal values for serum concentrations of total T3, T4, TSH Were: 1.26-2.76nmol/l, 57.9-161nmol/ml, and 0.4-4.0uIU/ml respectively.

Serum anti-thyroid peroxidase (TPO) antibody and anti-thyroglobulin antibody (TG): Was estimated by using (AESKULISA a-TPO kits, Germany) which is a solid phase enzyme immunoassay employing recombinant. Human Thyroid Peroxidase (TPO) from a eukaryotic expression system for the Quantitative and Qualitative detection of antibodies against TPO in human serum. In this study (in agreement with the ranges suggested by the manufacturer), the Normal range for anti TPO antibody was less than 87 IU/ml, border line (87-115) and the positive result was > 115 IU/ml. For anti-thyroglobulin antibody (TG); For anti thyroglobulin; The normal range anti bodies considered normal if Antibody titer less than 80IU/ml, border line if (80-100) IU/ml and Positive if antibody titer is more than 100IU/ml.

### Ethical Committee

This study was authorized by Ethical Committee, Issue No.5: Children Welfare Teaching Hospital, January 1st, 2017.

## RESULTS

This study included a total of 150 patients, with 100% response rate. The patients mean age and Std. Deviation was 9.72± 4.28. The most common age group was 5 - 10 year constituted 46% of the patients, and the lowest age category was <5 year constituted 12% of the patients.

For gender distribution, females constituted (52%) and (48%) were males. Most of the patients (66%) have less than three years duration of diabetes. Most of patients presented in this study had negative family history of autoimmune disease (77.3%). Regarding glycemic control, Only 23% of patients had strict glycemic control, while fair glycemic control constitute 36.7% of cases and 40% of patients had Poor glycemic control.

From the sera of 150 patients withType-1 diabetic patients; The positivity of thyroid Anti TPO was 17.3% of patients while Anti TG was positive in 28% of patients and both tests was positive in (11.6%) of patients. Based on autoantibody positivity and TSH concentration; TSH concentration was high in ten patients (6.6%) and autoimmune thyroid disease (AITD) were newly diagnosed only in three patients (2%).

According to distribution of thyroid AB with age group, the >10 year age group had the highest positive anti TPO AB and the <5 year age group had the lowest positive anti TPO AB but the result was not statistically significant (p=0.37). For anti TG AB the 5-10 year age group had the highest positive anti TG AB and the <5 year age group have the lowest positive anti TG AB titer but the result was not statistically significant (p=0.35).

The distribution of thyroid AB varies with the duration of DM. Although ≤3 year duration group had more positive anti TPO AB titer than >3 year group, the result was not statistically significant (p=0.15). (Table-I). Positive anti TG was more in ≤3 years duration group of DM and negative anti TG was less in the >3 years duration of DM group with statistically significant results (p=0.043) (Table-II).

**Table I:** Distribution of Anti TPO according to duration of DM, no. of patients 150.

Duration of DM AB		Duration of DM				p-value

		≤3 year		> 3 year		

		No.	%	No.	%	
Anti TPO	Positive	14	14%	12	24%	0.15
	Negative	85	86%	39	76%	

Total	99	100%	51	100%	

**Table II:** Distribution of Anti TG according to duration of DM, no. of patients 150.

Duration of DM AB		Duration of DM				p-value

		≤3 year		> 3 year		

		No.	%	No.	%	
Anti TG	Positive	33	33%	9	17%	0.043
	Negative	66	67%	42	83%	

Total	99	100%	51	100%	

Regarding the distribution of thyroid AB according to HBAC1 group, there was progressive positive anti TPO titer with glycemic status; Good glycemic control had the lowest positive anti TPO titer and poor glycemic control group had the highest positive anti TPO titer and the result was statistically significant (p=0.048), as shown in (Table-III).

**Table III:** Distribution of Anti TPO according to HBAC1 group, no. of patients 150.

Thyroid : AB		HBAC1 group						p-value

		Good glycemic control		Fair glycemic control		Poor glycemic control		

		No.	%	No.	%	No.	%	
Anti TPO	Positive	4	12%	6	11%	16	26%	0.048
	Negative	31	88%	49	89%	44	74%	

Total	35	100%	55	100%	60	100%	

Although anti TG titer also shows progressive increase with bad glycemic status. But result was not statistically significant (p=0.15) as shown in (Table-IV).

**Table IV:** Distribution of anti TG according to HBAC1 group, no. of patients 150.

Thyroid : AB		HBAC1 group						p-value

		Good glycemic control		Fair glycemic control		Poor glycemic control		

		No.	%	No.	%	No.	%	
anti TG	Positive	8	23%	12	22%	22	36%	0.15
	Negative	27	77%	43	78%	38	64%	

Total	35	100%	55	100%	60	100%	

### Statistical analysis

Statistical package for social science version 20 (SPSS 20) was used for both data entry and data analysis. Discrete variable presented as number (%). Chi-square test (or fisher exact test when appropriate) used to test the significance of association for discrete variable. P-value of < 0.05 was considered significant.

## DISCUSSION

The positivity for serum anti-TPO antibodies reported in this study was consistent with other studies.^9^-^14^ The lower percentage of positive Anti TPO than anti TG may be because that the anti TPO was more specific than Anti TGA.^15^ For this reason the Anti-TPO assayed by monoclonal anti body assisted RIA appears to be a more sensitive and specific marker for ATD than the conventional other tests.^15^ This also noted by Berg et al. in their study, and this was explained in that; the Anti TPO is associated with more elevation of TSH than ATG.^16^

A strong correlation exists between Thyroid-Auto antibodies and the future risk of thyroid dysfunction in patients with Type-1 diabetes.^17^ Based on autoantibody positivity and TSH concentration, TSH concentration was high in 16% of the studied patients and all of them had positive auto immunity. While an overt thyroid dysfunction was observed only in three female patients with hypothyroidism, this is in consistent with other literatures which showed that ATD more often produces signs of hypothyroidism (Hashimoto’s thyroiditis) and less frequently of hyperthyroidism (Graves’ disease or the hyperactive phase of Hashimoto’s thyroiditis).^18^,^19^ Furthermore, the long period of autoimmunity makes the clinical diagnosis of thyroid autoimmune disease difficult because it often runs in subclinical state. Measurements of thyroid functions tests in clinically suspected cases or in patients with positive Thyroid antibodies (TAA) would confirm the disease.^20^ Although positive anti TPO was the same in both duration group of DM, the less 3 year group shows more positive anti TG titer with statistically significant results (p=0.043). This is comparable with other reports^21^, which showed a decrease in the concentrations of thyroid antibodies with increasing duration of diabetes, this may be explained in part by increase seroconversion peaks of thyroid antibodies around the puberty, at which time the thyroid gland undergoes remodeling.^22^ In this study, there is significant correlation between positive result of serum antibodies and poor glycemic control. The associated autoimmune disease may influence the control of diabetes by impairing function of the respective organ. Moreover, thyreotoxi-cosis may worsen the metabolic control of diabetes and increase the need for increased insulin dosage, and hypothyroidism can lead to increased frequency of hypoglycemic episodes in diabetic patients.^22^,^23^ Metwalley KA et al. in their study in Egypt showed that Diabetic patients with hypothyroidism had higher HbA1c than those without hypothyroidism.^24^

## CONCLUSION

The screening of autoantibodies in Type-1 diabetic patients could reveal subclinical cases AITD, but its predictive value for progression to clinical manifestation is limited. The follow-up of patients with positive autoantibodies is necessary because further deterioration of diabetic control and dysfunction of the respective organs may occur.

### Author`s Contribution

**MF:** Conceived, designed, statistical analysis, editing of manuscript, data collection and manuscript writing.

**MZ:** did review and final approval of manuscript.
